# The Human Microbiome and Infectious Diseases: Beyond Koch

**DOI:** 10.1155/2008/296873

**Published:** 2009-03-29

**Authors:** Vincent B. Young, Robert A. Britton, Thomas M. Schmidt

**Affiliations:** ^1^Division of Infectious Disease, Department of Internal Medicine, University of Michigan, MI 48109, USA; ^2^Department of Microbiology and Immunology, University of Michigan, MI 48109, USA; ^3^Microbiology and Molecular Genetics, Michigan State University, MI 48824-4320, USA

A century after Robert Koch linked individual cultured microbes to specific diseases (Koch's postulates), it has become increasingly apparent that the complex community of microorganisms associated with the human body plays a key role in health and disease. The National Institutes of Health recently announced the Human Microbiome Project as part of the NIH Roadmap for medical research, with a primary goal of advancing our understanding of the relationships among host-associated microbial communities, health, and disease. Many physicians and researchers, however, have only passing familiarity with the concepts involved in the study and therapeutic manipulation of complex microbial communities.

This special issue was conceived to accomplish several goals. We wanted to provide readily accessible overviews of the concepts and methods used in the study of complex microbial communities, and demonstrate how changes in indigenous microbial communities can play a role in diseases such as antibiotic-associated diarrhea and bacterial vaginosis. We also set out to find examples of how probiotics can be used for the therapeutic manipulation of the indigenous microbiota.

We were fortunate to receive a strong collection of review articles and primary research manuscripts to meet the goals of this special issue. In the first article “Conceptualizing the human microbiota: from multicelled organ to ecological community,” B. Foxman et al. present a novel conceptualization of the human microbiome that blends perspectives of epidemiologists, classical ecologists and infectious diseases physicians. The second article “Application of ecological network theory to the human microbiome,” by J. Foster et al., outlines how ecological network theory can be applied to studies of the human microbiome, while the third article “Interactions of the intestinal epithelium with the pathogen and the indigenous microbiota: a three way crosstalk,” by C. V. Srikanth and B. McCormick, review the interactions between epithelial pathogens and the indigenous microbiota in the mammalian gut. In the fourth article “Application of sequence-dependent electrophoresis fingerprinting in exploring biodiversity and population dynamics of human intestinal microbiota: what can be revealed?” G. Huys et al. review the use of sequence-dependent fingerprinting methods for studying the structure and dynamics of complex microbial systems using the human intestinal microbiota as an example. The fifth article “Ecological characterization of the colonic microbiota of normal and diarrheic dogs,” by J. Bell, employs such a fingerprinting method to study the canine colonic microbiota. The sixth article “Emerging insights into antibiotic-associated diarrhea and *Clostridium difficile* infection through the lens of microbial ecology,” by S. Walk and V. Young, discusses the role of the gut microbiota in antibiotic-associated diarrhea and *Clostridium difficile* infection while Y. Sanz et al., in the seventh article “Insights into the roles of gut microbes in obesity,” review the evidence for the role of gut microbes in obesity. In the eighth article “The human vaginal bacterial biota and bacterial vaginosis,” by S. Srinivasan and D. Fredericks review the human vaginal bacterial microbiota and the ninth article “Temporal shifts in microbial communities in non-pregnant african-american women with and without bacterial vaginosis,” by J. Wertz et al., examines this microbial community in the setting of bacterial vaginosis. The tenth article “Vaginal microbiota and the use of probiotics,” by S. Cribby et al. discusses the use of probiotics to alter the vaginal microbiota. Finally, the eleventh article “Probiotics and gastrointestinal infections,” by R. Britton and J. Versalovic, and the twelveth article “Probiotic bacteria influence the composition and function of the intestinal microbiota,” by P. O’Toole and J. Cooney, summarize the potential role of probiotics to influence gastrointestinal health.

